# Emergency room use after being released from incarceration

**DOI:** 10.1186/2194-7899-2-5

**Published:** 2014-03-25

**Authors:** Erlyana Erlyana, Dennis G Fisher, Grace L Reynolds

**Affiliations:** 1 Health Care Administration Department, California State University Long Beach, 1250 Bellflower Blvd.,, Long Beach, CA 90840 USA; 2 Center for Behavioral Research and Services, California State University Long Beach, 1090 Atlantic Avenue, Long Beach, CA 90813 USA; 3 Center for Behavioral Research and Services, California State University Long Beach, 1250 Bellflower Blvd.,, Long Beach, CA 90840 USA

**Keywords:** Emergency room, Use, Opiate use, Incarceration, Mediation

## Abstract

**Background:**

This study investigates opiate use in mediating the impact of history of incarceration on emergency department (ED) use.

**Method:**

Data were collected from 1,341 clients who underwent HIV and STI screening in an outpatient care center in Long Beach, California. The Risk Behavior Assessment (RBA, baseline) and Risk Behavior Follow-Up Assessment (RBFA, follow-up) were administered to each client with at least a three months interval between assessments.

**Results:**

Of 1341 participants in the study, 931 (69.43%) reported previous incarceration. Having a history of incarceration was significantly associated with emergency room use as well as a history of sexually transmitted infections (STIs), injection drug use, opiate use, and survival sex trading, defined as sex for money or drugs. The relationship between previous incarceration and ED use was mediated by opiate use for men but not for women. The findings suggested that the effect of history of incarceration on ED use was exacerbated when the individuals were male and opiate users.

**Conclusions:**

Targeted interventions in treatment and rehabilitation programs could help prevent unnecessary ED use and reduce the use of EDs through drug treatment during incarceration and after release.

**Electronic supplementary material:**

The online version of this article (doi:10.1186/2194-7899-2-5) contains supplementary material, which is available to authorized users.

## Background

Providing adequate support for adjustment after being released from incarceration remains a public health challenge. The magnitude of the unmet health needs and services gaps for inmates and ex-prisoners has been under-studied (Mears & Cochran, 2012). Uninterrupted access to adequate health care may alleviate negative social and health consequences for former inmates, and improve the success of their re-entry to society (Freudenberg et al. 2008; Wang et al. 2012). Receiving continuing and longer periods of substance abuse treatment after being released from prison was positively associated with greater success on parole (LaMoure et al. 2010). In the long run, adequate access to health care may reduce the misuse and spending associated with emergency room (ED) visits for non-emergency cases. Therefore, it is important to investigate patterns of ED use by reentry populations, particularly factors that mediate the use of EDs among individuals with a history of incarceration.

## Literature review

Medical care delivered in an ED plays a significant role in the US health care system (it consumed 4% of all health care spending in 2010). National data show that an average of 20% of the US population visits an ED each year (Owens et al. 2010) and the total number of visits to EDs increased 34% from 97 million in 1995 to 130 million in 2010 (a 16% increase from 37 visits per 100 population to 43 visit per 100 population) while ED supply decreased by 11% (NCHS, 2013). The ED serves as an entry point for inpatient admissions and is a common setting for acute care. Major challenges, however, have been ED overcrowding, and use of the EDs for non-emergency care by uninsured and underinsured individuals who lack access to primary care providers. California with about 10 million emergency room visits annually (McConville & Lee, 2008), is potentially facing a bigger challenge with the ruling of the US Supreme Court to reduce overcrowding in California prisons.

Several studies reported that individuals with a history incarceration were more likely to have poor health outcomes, high-cost health services use, and low use of regular medical services. A history of incarceration has been associated with a higher prevalence of infectious disease including HIV and STIs; mental illness; and substance abuse and dependency (Davis & Pacchiana, 2004; Fazel et al. 2006; Fisher et al. 2006; Wilper et al. 2009); a higher prevalence of chronic diseases (Binswanger et al. 2009; Davis & Pacchiana, 2004; Hawkins et al. 2010; Wang & Green, 2010), and a higher risk of death (Binswanger et al. 2007). The poor health status of reentry populations also led to use of high-cost health services (Leukefeld et al. 2006). Longer and more severe criminal history was associated with poorer physical health and use of the ED (Mateyoke-Scrivner et al. 2003). Previous studies have shown that the ED was the primary source of care among released prisoners with HIV (Meyer et al. 2012), and drug-involved prisoners (van Olphen et al. 2006). Challenges in accessing health care and medication and risk factors for HIV and hepatitis C infection (HCV) were more prevalent among former inmates, especially immediately after release (Adams et al. 2011; Hawkins et al. 2010; Kulkarni et al. 2010). The long term effects of incarceration increases the likelihood of poor health (Schnittker & John, 2007).

Prevalence of substance use and dependence was higher among prisoners than the general population, particularly among drug using women (Fazel et al. 2006). Incarceration and drug use prior to incarceration have been associated with injection drug use among inmates (Calzavara et al. 2003). Prisons may be high-risk environments for the initiation and use of heroin and other drugs (Boys et al. 2002). In addition to being an ongoing challenge for corrections, drug abuse may predispose an individual to misuse or underutilize health services (Leukefeld et al. 1998). Drug abusers were more likely to use the ED (Cherpitel & Ye, 2008; Gilbert et al. 2012), have higher medical expenses than non-drug abusers (French et al. 2000), and a higher risk of death (Merrall et al. 2010). Illnesses, poorer health status, and use of physical and mental health services were also more common among prisoners who were drug abusers than among non-drug abusing prisoners (Garrity et al. 2002; Kanato, 2008; Webster et al. 2005). The literature also suggests that only heavy illicit drug users were reported to use EDs rather than non-drug users (French et al. 2011) and longer and more frequent drug users were more likely to have unmet health care needs (Narevic et al. 2006). Injection drug users were more likely to visit an ED than non IDUs (Kerr et al. 2005) and frequent drug injectors were more likely to use an ED (Marshall et al. 2012; Stein & Anderson, 2003). Among women, stigma caused by drug use and incarceration increases the need for health and social services, but restricts access to those services (van Olphen et al. 2009).

Individuals with a history of incarceration (former inmates) were more likely to engage in risky sexual behavior (unprotected sex and transactional sex), immediately upon release (Adams et al. 2011). Arrest and incarceration are temporally associated with and contribute to sexually risky behavior (Epperson et al., 2009). Incarceration was associated with high-risk sex partnerships and acquisition of sexually transmitted infections (STI) and human immunodeficiency virus (HIV; (Fisher et al. 2004; Jenness et al. 2011; Khan et al. 2009; Weiser et al. 2006). Sex trade involvement was associated with having abscesses among injection drug users (Lloyd-Smith et al. 2005) and due to occupational stigma, sex workers were reported to lack access to health care (Lazarus et al. 2011). Other barriers to receiving health care include structural (poverty, homelessness) and individual factors such as drug use, fear of violent victimization, and mental illness (Kurtz et al. 2005). Having multiple sex partners was a significant predictor of ED visits (El-Bassel et al. 2007). This study investigates whether the impact of history of incarceration on ED use is mediated by opiate use and sex trading for drugs or money, two current public health problems with substantial risks to individual health.

## Methods

### Datasets

Data were collected from clients who presented for HIV and STD screening in an outpatient care center in Long Beach, California. Each client was interviewed using two different instruments: the Risk Behavior Assessment (RBA, baseline), and the Risk Behavior Follow-Up Assessment (RBFA, follow-up) with at least a three-month interval between administration of the RBA and RBFA. The RBA elicits information on drug utilization (use of any type of drugs in the last month, use of injected drugs, and frequency of use), sex trading practices, and history of incarceration (including length of time in jail or prison). The RBA has been shown to have good reliability and validity (Dowling-Guyer et al. 1994; Fisher et al. 2004). The RBFA elicits information on use of an emergency room in the last three months and number of hospitalization days during that period (Johnson et al. 2000). Other information collected included age, gender, sexual orientation, race, marital status, educational attainment, living arrangements, homelessness, and income. The RBA-RBFA system was originally developed by grantees funded by the National Institute on Drug Abuse (NIDA) along with NIDA staff from the Community Research Branch. There were 23 grantees in this NIDA Cooperative Agreement. Most of the Cooperative Agreement data used a 6-month follow-up period, but did not find any differences between conditions at follow-up. Because of this, the sites that were funded in the last round of funding developed a 3-month version of the RBFA to better detect differences that might occur in this shorter time period, but might dissipate on longer follow-up. Two of the authors on the current study have been very interested in this question of 3 versus 6 month recall period and published a meta-analysis on this topic (Napper et al. 2010). In this meta-analysis, it was found that the reliabilities for most sex behaviors and most drug use were better with the shorter recall period. Therefore this study used the 3-month recall period to maximize reliability.

### Participants

The present study recruited participants through the Center for Behavioral Research and Services (CBRS) in Long Beach, California. In order to be eligible, participants had to have been at least 18 years of age at the time of interview and have photo identification, which also included their date of birth. The study used a sample of participants that come to CBRS who wish to be tested for HIV, hepatitis B, hepatitis C, and/or syphilis. The sample also consisted of participants recruited through snowball sampling, as many participants refer others into testing. Additionally, recruitment came from other programs in the community encouraging HIV and STD testing. Such programs included drug and alcohol treatment programs including the Substance Abuse Foundation (SAF) located in Long Beach. Interviews were conducted in private offices to ensure confidentiality and instruments were administered by trained interviewers. Participants were provided with a $5 non cash incentive in the form of a gift card for their participation. All study procedures were approved by the Institutional Review Board at California State University, Long Beach.

### Variables

The dependent variable is a dichotomous variable of whether the respondents ever used an emergency room in the last 3 months for any reason. The potential independent variables, collected from the RBA, were whether the respondents have ever been in jail or prison for any reason and length of time spent in a jail or prison. The potential mediator variables included use of specific types of drugs (marijuana, opiates such as heroin and non-prescription methadone, crack, cocaine, amphetamine) in the last 30 days, use of injected drugs, and whether the respondent had ever traded sex for drugs or money. Previous work has indicated that these variables are associated with health services usage among at-risk individuals in Los Angeles County (Erlyana, et al. 2014 in press) especially those who access publicly funded HIV and sexually transmitted diseases testing services (Fisher, et al. 2010).

### Statistical analysis

**Figure 1:**
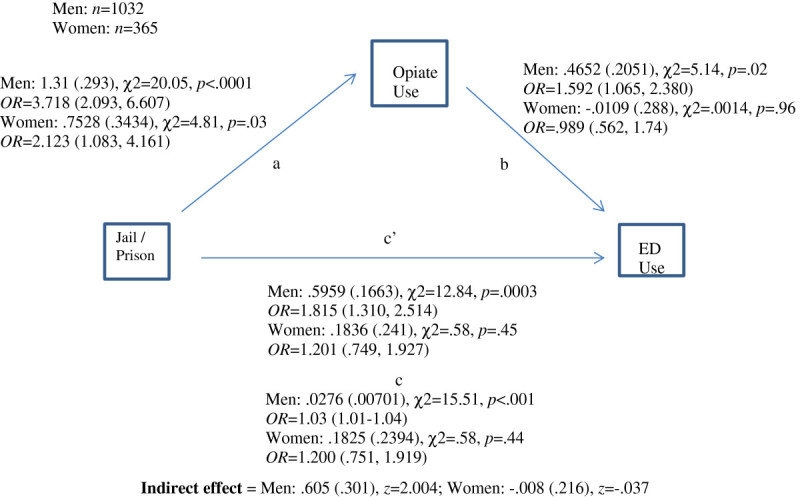
**Path diagram for mediation model of emergency room use, previous incarceration and opiate use by gender.**

This study utilized mediation analysis to investigate factors that mediated the relationship between history of incarceration and ED use. Use of mediation analysis explains the mechanism through which the independent variable affects the dependent variable (MacKinnon, 2008). A single mediator model determines whether the relationship of the independent variable to the dependent variable was mediated by another third variable. Logistic regression was used because the response (outcome variable) of emergency room use was a binary variable coded at 1 = any ED use in the past 3 months or 0 = no ED use in the past three months. Mediation models are developed through a series of regression models where the direct effect of the independent variable are assessed by regression coefficients and standard errors; the effect of the mediator variable is also assessed by regression coefficients and standard errors; and the indirect or mediated effect is of the independent variable on the outcome variable via the mediator variable is then calculated. Because men and women have different patterns of health care use with women consistently reporting more frequent use compared to men, the mediation models were done separately by gender. The mediator variables that were tested in the current study included whether the respondents used opiates or whether the respondents had ever traded sex for money/ drugs. We hypothesized that respondents who used opiates were more likely to use the ED. We also hypothesized that those who traded sex for drugs or money were also more likely to use an ED. We employed the notation used by MacKinnon (2008) (see Figure 1) where c is the direct effect of the IV on the outcome; b is the effect of the mediator variable on the outcome; a is the effect of the IV on the mediator, and c’ is the mediated effect of the IV on the outcome via the mediator variable. A *z* score is then calculated to determine the statistical significance of the mediated or indirect effect. All analyses were conducted in SAS 9.3.

## Results

### Descriptive statistics

Of the 1341 participants who completed both the RBA and RBFA, 417 (31.1%) indicated that they had used an emergency room for any reason at least once in the last three months. Compared to those who did not report use of the ED, those who reported use of the ED were more likely to have a history of incarceration (76.26% vs. 66.34%, χ^2^ (1) = 13.31, *p* = .0003), a longer average time in jail or prison (3.75 years vs. 3.06 years, *t* (1243) = -1.88, *p* = .0604), and to have ever traded sex for money or drugs (38.73% vs. 29.01%, χ^2^ (1) = 12.68, *p* = .0004). Those who reported use of the ED were also more likely to be opiate users (16.78% vs. 11.73%, χ^2^ (1) = 6.52, *p* = .0107) and to have ever received drug treatment (60.42% vs. 48.56%, χ^2^ (1) = 16.54, *p* < .0001) than those who did not use an ED (Table 1). The hypothesized relationship of sex trading as a mediator of ED use was not significant for men or women.

**Table 1:** **Characteristics of ED users vs. non- ED users**

Variables	Has ever use ED	Never use ED	***t***, df or χ^2^( ***df***)	***p-*** value
	***n*** = 417	***n*** = 924		
	Mean (SD) or%	Mean (SD) or%		
Age	40.8 (11.21)	40.5 (11.59)	-.47, 1338	.6381
Race				
Black	45.19	43.29	5.33 (3)	.1488
Whites	33.17	29.33		
Hispanics	14.66	18.72		
Others	6.97	8.66		
Has a history of incarceration	76.26	66.34	13.31 (1)	.0003
Has ever had STIs*	49.53	40.30	10.21 (1)	.0014
Has ever traded sex for drugs or money	38.73	29.01	12.68 (1)	.0004
Opiate use	16.78	11.73	6.52 (1)	.0107
Injection drug use	28.81	23.13	5.05 (1)	.0247
Income				
< $1,000	78.77	66.71	18.18 (2)	.0001
$1,000 -$3,999	18.49	24.57		
> $3,999	2.74	8.71		
Had paid job, salary, or business	21.5	31.92	15.14 (1)	.0001
On welfare, public assistance	39.23	32.24	6.15 (1)	.0131
Had Social Security, disability	30.99	20.78	16.31	.0001
Heterosexual	67.69	61.59	4.68 (1)	.0305
Marital status				
Single	53.63	63.68	13.49 (2)	.0012
Married	20.14	17.41		
Separated/divorced	26.23	18.91		
Education				
Less than High School	29.84	27.19	6.64 (2)	.0361
High School	37.53	32.94		
Some college or above	32.63	39.87		
Homeless	47.04	41.04	4.28 (1)	.0386
Female	32.08	22.95	12.79 (1)	.0003
Has ever received drug treatment	60.42	48.56	16.54 (1)	.0001

Note: *include hepatitis B, gonorrhea, syphilis, genital warts (HPV), chlamydia, and genital herpesMen: .5959 (.1663), χ2 = 12.84, *p* = .0003.

### Mediation analysis

Of 1341 participants of the study, 931 (69.43%) reported previous incarceration. Figure 1 presents the model of how the effect of past history of incarceration on subsequent ED use is mediated by opiate use. The hypothesis was that those with a history of incarceration were more likely to use opiates, which then led to ED use. Men with a history of incarceration were significantly more likely to use an ED (ĉ = .0276, s_ĉ_ = .0071, χ^2^ = 15.51, OR = 1.03). There was a statistically significant effect of history of incarceration on opiate use for men (*a* = 1.31 (.293), χ^2^ = 20.05, *p* < .0001, OR = 3.718) and women (*a* = .7528 (.3434), χ^2^ = 4.81, *p* = .03, OR = 2.123) however the opiate use was significantly associated with ED use for men only (b = .4652 (.2051), χ^2^ = 5.14, *p* = .02, OR = 1.592). The adjusted effect of past history of incarceration was statistically significant for men (c’ = .5959 (.1663), χ^2^ = 12.84, *p* = .0003, OR = 1.815) but not for women. This means that the opiate use mediated the effect of past history of incarceration on ED use for men. The total mediated effect (indirect effect) of opiate use on past history of incarceration and ED use was significant for men but not for women (*âb* (std. err.) = .605 (.301), *z* = 2.004 (see Figure 1).

## Discussion

This study explored models to determine whether the use of emergency room services post-incarceration is mediated in a meaningful way by a third variable for men or women. As noted, the relationship between history of incarceration and ED use was significant for men but not for women in our sample, in that those with a history of incarceration were more likely to report use of the ED (Figure 1). The relationship for men was mediated by opiate use which suggests that ED use was more prevalent among male opiate-using former prisoners (van Olphen et al. 2006) and heavy illicit drug use has previously been identified as a significant predictor of ED use (French et al. 2011). History of incarceration was significantly associated with opiate use for both men and women, however, from the current study we do not know if drug use occurred only in jail/prison, only during those periods when the participants were not incarcerated, or both. While incarceration was significantly associated with injection drug use, the Anderson model posits multiple vulnerabilities (Calzavara et al. 2003) as pathways to health services usage. The total mediated effect (indirect effect) of opiate use in this study was significant only for men suggesting that opiate use is a vulnerability for men but not for women in terms of ED use.

The relationship between history of incarceration and ED use was not mediated by sex trading for drugs or money for either men or women (data not shown). This was true for sex trading for money or drugs as well as a combination variable of survival sex, which included trading sex for both money and drugs.

This study did find that a history of incarceration was significantly associated with sex trading for drugs or money as suggested by the literature, whereby former inmates were found to be more likely to engage in transactional sex immediately upon release from prisons (Adams et al. 2011) and to engage in high-risk sexual partnerships (Jenness et al. 2011). The study also found that those who traded sex for drugs or money were significantly more likely to report ED use as the literature has reported that sex workers, due to occupational stigma and fear of violent victimization, were less likely to have access to primary health care (Kurtz et al. 2005; Lazarus et al. 2011). However, these relationships, while significant in bivariate analyses were not significant for either men or women when sex trading for either drugs or money, or both, was used in a mediation model.

There are several limitations that need to be noted in this study. First, there is a need to capture information on reasons for use of the ED. This information would be particularly useful to separate cases that are episodes of non-acute medical needs from assault-related or drug-involved incidents, such as overdose. Data on having a regular source of care (outpatient care such as a primary care physician) and health status of the respondents were not available. The current study did not capture variables associated with mental health status, including depression and anxiety, which may influence use of emergency rooms (Sandoval et al. 2010). This study focused on out-of-treatment drug users whose opiate use consisted mainly of heroin and non-prescription methadone. The majority were low income, reporting $1,000 or less per month. The findings reported here may not generalize to individuals who are misusing prescription opiates, although it has been suggested in other studies (Reynolds et al. 2000). Lastly, the sample used in this study has a relatively higher rate of ED use compared to the general population (30% vs. 20%), and most probably is due to an extremely high prevalence of individuals with a history of incarceration (about 70% of the total sample).

## Conclusion

Future research comparing ED and outpatient care use for previously incarcerated individuals should consider mediation models that control for the health status of the respondents. Continued access to Medicaid (Wakeman et al. 2009) and engaging individuals to use primary care in transition clinics (Wang et al. 2012) should be considered as alternatives to reduce societal burden associated with increasing spending and overcrowding of EDs.

## Authors’ original submitted files for images

Below are the links to the authors’ original submitted files for images. Authors’ original file for figure 1
