# Supplementary figures and images for: Emergency room use after being released from incarceration
Source: Health Justice. 2014 Mar 25;2:5. doi: 10.1186/2194-7899-2-5 (PMC5151729; doi:10.1186/2194-7899-2-5)

Men:  $n=1032$

Women:  $n=365$

Men: 1.31 (.293),  $\chi^2=20.05$ ,  $p<.0001$

OR=3.718 (2.093, 6.607)

Women: .7528 (.3434),  $\chi^2=4.81$ ,  $p=.03$

OR=2.123 (1.083, 4.161)

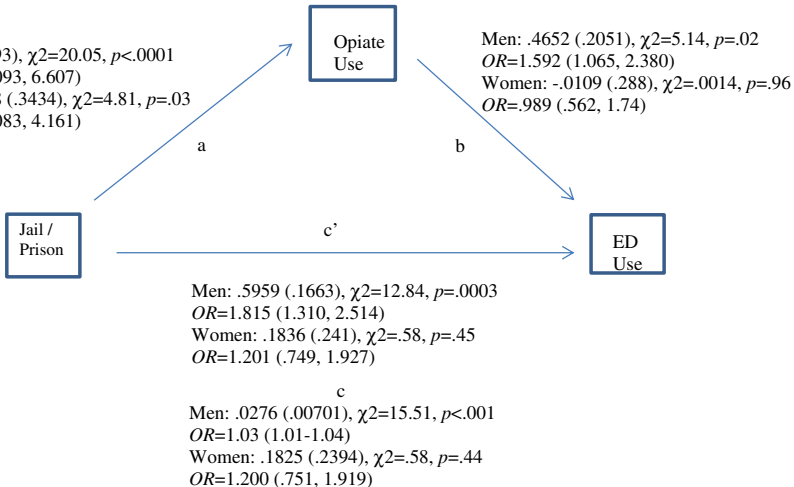

**Indirect effect** = Men: .605 (.301),  $z=2.004$ ; Women: -.008 (.216),  $z=-.037$

Supplement: Supplementary file 1 — Authors’ original file for figure 1 [file 40352_2013_14_MOESM1_ESM.pdf]
